# Genetic Polymorphisms in the Apoptosis-Associated Gene *CASP3* and the Risk of Lung Cancer in Chinese Population

**DOI:** 10.1371/journal.pone.0164358

**Published:** 2016-10-10

**Authors:** Jia Lin, Yanyan Zhang, Hongge Wang, Jiang Chang, Lixuan Wei, Lei Cao, Zhi Zhang, Xuemei Zhang

**Affiliations:** 1 Department of Molecular Genetics, College of Life Science, North China University of Science and Technology, Tangshan, China; 2 Department of Epidemiology, School of Public Health, North China University of Science and Technology, Tangshan, China; 3 Department of Epidemiology and Biostatistics, and State Key Laboratory of Environment Health (Incubation), MOE (Ministry of Education) Key Laboratory of Environment & Health, Ministry of Environmental Protection Key Laboratory of Environment and Health (Wuhan), School of Public Health, Tongji Medical College, Huazhong University of Science and Technology, Wuhan, China; 4 Department of Etiology and Carcinogenesis, Cancer Institute and Hospital, Chinese Academy of Medical Sciences and Peking Union Medical College, Beijing, China; 5 Department of Chemotherapy and Radiotherapy, Tangshan Gongren Hospital, Tangshan, China; Duke Cancer Institute, UNITED STATES

## Abstract

Caspase-3 (CASP3) plays a central role in executing cell apoptosis and thus in carcinogenesis. We previously investigated the relationship between functional polymorphisms in *CAPS3* 829 A>C and 20541 C>T and risk of esophageal squamous cell carcinoma. However little is known about the role of *CASP3* variants in susceptibility to lung cancer. To figure out the contribution of *CASP3* polymorphisms to lung cancer risk, genotypes of 1000 lung cancer patients and 1000 controls were conducted by RFLP-PCR (restriction fragment length polymorphism PCR). The transcriptional activity of CASP3 829 A>C was examined by dual luciferase reporter assay. Logistic regression was applied to calculate Odds ratios (OR) and 95% confidence intervals (95%CI). Compared with *CASP3* 829 AA genotype, AC and CC genotype had significantly increased risk of lung cancer with OR (95% CI) of 1.33 (1.09–1.63) and 1.55 (1.19–2.01), respectively. To further explore the possible impact of 829 A>C SNP on *CASP3* transcriptional activity, we detected the dual luciferase activity of PGL3-promoter vectors containing 829A or 829C alleles in lung cancer cell lines and found that report gene expressions driven by 829A containing *CASP3* promoter were 1.64-fold, 1.94-fold greater than those driven by *CASP3* 829C containing counterparts in A549 and NCI-H1975 cells (P<0.001). When stratified by sex, the significantly increased risk associated with *CASP3* 829 AC or CC genotype was obviousl in males with OR (95% CI) of 1.42 (1.11–1.81) and 1.51 (1.11–2.05), but not in females. When stratified by age, we found that *CASP3* 829 AC or CC genotype contributed to the risk of lung cancer in youngers with OR (95% CI) of 2.73 (1.71–4.34) and 4.02 (2.20–7.32), but not in elder group. We also found that 829AC or 829CC genotype increased adenocarcinoma risk compared with the AA genotype with OR (95%CI) of 1.33 (1.04–1.70) and 1.51(1.09–2.07). *CASP3* polymorphism and smoking interaction was demonstrated related with higher risk of lung cancer. We achieved that the *CASP3* 829AC or 829CC genotypes was associated with increased risk of lung cancer in both non-smoker and smoker group, with OR (95%CI) of 1.48 (1.08–2.02) and OR (95%CI) of 1.64 (1.09–2.48) among non-smokers and OR (95%CI) of 2.68 (1.89–3.81) and OR (95%CI) of 3.23 (2.21–4.92) among smokers, respectively. Among carriers with 20541CT genotype, the ORs (95%CI) of risk with lung cancer for smoking <16, 16–28, or > 28 pack-years were 1.16(0.65–2.07), 1.66(0.98–2.82) and 5.01(3.31–7.58) compared with the 20541CC carriers. And among carriers with 20541CT genotype, the ORs (95%CI) were 0.86(0.33–2.20), 2.12(0.83–5.41) and 5.71(2.68–12.16). These results highlight apoptosis-related *CASP3* as an important gene in human carcinogenesis and further support the *CASP3* polymorphisms confer to the lung cancer susceptibility.

## Introduction

Lung cancer is a malignant lung tumor and leads to massive death worldwide. Many factors including tobacco smoking, living habit, environmental and eating factors are vital causes of lung cancer [1]. We all have known that smoking is a major factor for lung cancer, but only some of smokers suffer form lung cancer through lifetime. It is concluded that gene differences of each individual partly determine the susceptibility to lung cancer [2]. Thus, we develop a further study to discover the molecular gene markers which can give rise to the high risk developing lung cancer.

Along the apoptosis process, some kinds of death proteases are activated and cell changes biochemically and morphologically [3, 4]. Therefore apoptosis may cause the somatic mutations and now thought to contribute to a number of human diseases, ranging from neurodegenerative disorders to malignancy [5, 6]. The dislocation of apoptosis contributes to tumor development and progression [7]. Caspases (CASPs) is a kind of cysteine-dependent aspartate-specific proteases, and in charge of the initiation and execution of apoptosis. Based on their functions, CASPs can be devided into initiator CASPs and effector CASPs based on their proapoptotic functions. CASP8, CASP9, and CASP10 belong to initiator CASPs, and they transmit apoptotic signals; CASP3, CASP6, and CASP7 belong to activate effector CASPs, and they perform the final cell death process [3]. Caspase-3 (CASP3) plays an essential role during apoptotic cell death by proteolytic cleaving a variety of key proteins required for cellular functioning and survival [8]. PARP-1 (poly ADP-ribose polymerase 1) is one main substrate of CASP3. When apoptosis begins, CASP3 cleaves PARP-1 into two fragments to inactivate the enzymatic activity of PARP-1. It increases the activity of one kind of endonucleases which can induce cell apoptosis though DNA cleaving [8].

Takata et al. indicated that caspase-3 was expressed in both the nucleus and the cytoplasm of lung cancer cells [9]. *CASP3* mutations were detected many types of tumor, including colon carcinomas, non-small cell lung cancers, non-Hodgkin lymphomas, stomach carcinomas, hepatocellular carcinomas, and multiple myelomas [10]. Xie et al. sequenced 261 DNA samples from healthy individuals of Han Chinese population to search for genetic variants within the regulatory region, exons 2–7 and their flanking sequences of CASP3. They identified three single nucleotide polymorphisms (SNPs), 829 A>C, 17532 A>C, and 20541 C>T, which located in 5’-regulatory region, intron 4, and 3’-regulatory region of CASP3, respectively. They also found that 17532 A>C and 20541 C>T were in complete linkage disequilibrium [11]. Based on these, we final investigated CASP3 829 A>C and 20541 C>T polymorphisms in this lung cancer case-control study.

## Materials and Methods

### Study subjects

Our case-control study collected 1000 lung cancer patients and 1000 healthy controls. Keep all participators genetically unrelated ethnic Han Chinese. All the cases were newly diagnosed, histopathologically confirmed, and previously untreated (by radiotherapy or chemotherapy) primary lung cancer. The patients were recruited between January 2008 and December 2012 at Tangshan Gongren Hospital. There were no age, sex, stage, or histology restrictions; however, patients with previous cancer or metastasized cancer from other organs were excluded. The controls were randomly selected from a pool of cancer-free subjects recruited from a nutritional survey conducted in the same region during the same period as the cases were collected. The selection criteria include no prior history of cancer, and controls were matched to the cases by age (±5 years) and sex. At recruitment, informed consent was obtained from each subject who was then interviewed for detailed information on demographic characteristics and lifetime history of tobacco use. The study was approved by the institutional review board of North China University of Science and Technology. All participants provide their written informed consent to participate in this study.

### Genotype Analysis

**Fig 1:**
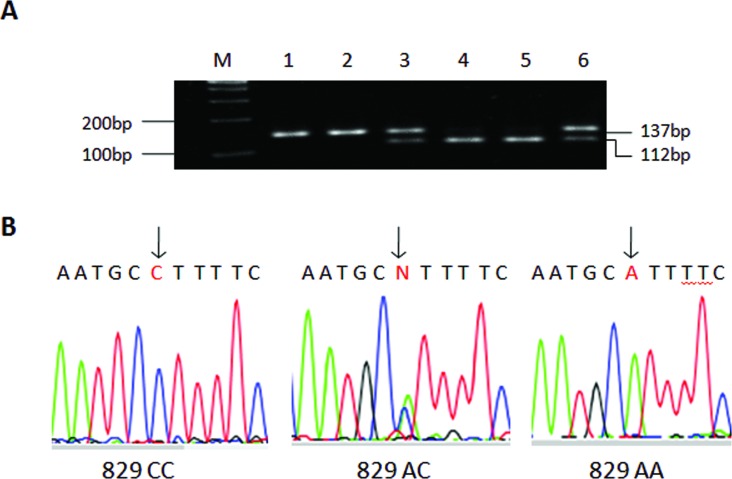
Analysis of the *CASP3* 829 A>C polymorphism. A, representative gel picture showing PCR-RFLP analysis of the *CASP3* 829 A>C genotypes in genomic DNAs of study subjects with the restriction enzymeBgl*I*. M, DNA size markers; subjects 4 and 6, AA genotype; subjects 2 and 3, CC genotype; subjects 1 and 5, AC genotype. B, partial DNA sequence of three different allelic PCR products analyzed directly with an ABI PRISM 377 automatic sequencer showing a Ato Ctransversion at the nucleotide location at which the arrow point.

**Fig 2:**
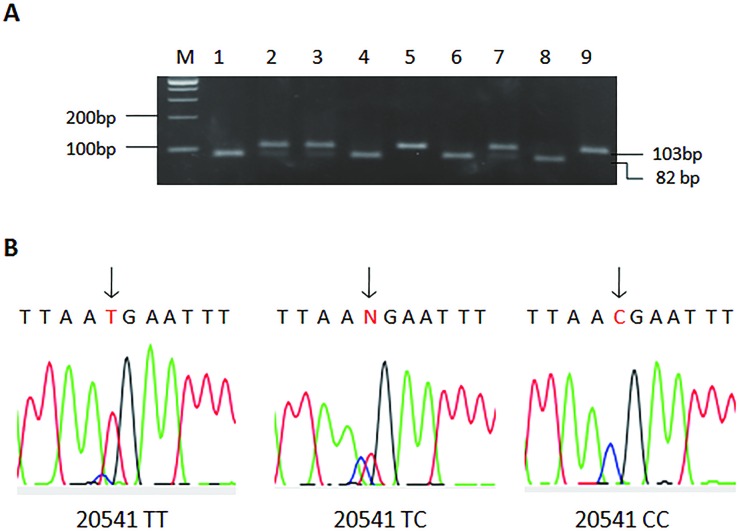
Analysis of the *CASP3* 20541 C>T poly-morphism. A, representative gel picture showing PCR-RFLP analysis of the *CASP3* 20541 C>T genotypes in genomic DNAs of study subjects with the restriction enzyme Ase*I*. M, DNA size markers; subjects 1, 4, 6, 8, CC genotype; subjects 2, 3, 7, CT genotype; subjects 5 and 9, TT genotype. B, partial DNA sequence of three different allelic PCR products analyzed directly with an ABI PRISM 377 automatic sequencer showing a C to Ttransversion at the nucleotide location at which the arrow point.

Genomic DNA of all controls and patients was extracted from peripheral blood lymphocytes. RFLP-PCR (restriction fragment length polymorphism PCR) analysis was applied for genotyping the *CASP3* SNPs. Briefly, to produce *CASP3* region containing the 829 A>C (rs4647602) site, the PCR primer pairs was 5’-TAG TTG CAG GGT TTA AAC TCC AAT GC-3’ and 5’-CTA ACT CCT CAC GGC CTG GGA T-3’. The primer pairs used to amplify CASP3 20541 C>T (rs1049216) was 5’-GTG AAA AAG TTA AAC ATT GAA TTA A-3’ and 5’-TTC TTC CAC ATC ATC ATT TCT A-3’. The two primer pairs were coincident in our previous epidemiological study of esophageal squamous cell carcinoma [12]. In brief, PCR was performed using a 25-μl reaction mixture containing 100 ng DNA, 0.1 μmol⁄/L each primer, 0.2 mmol/L deoxynucleoside triphosphate, and 1.0 U Taq DNA polymerase (TaKaRa). The PCR profile consisted of an initial melting step of 95°C for 4 min, followed by 35 cycles 94°C for 30 s, 60°C for 30 s, 72°C for 30 s, and a final extension step of 72°C for 7 min. The amplified PCR products for 829 A>C (137bp) and 20541 C>T (103bp) were digest with Bgl*I* and Ase*I* (New England Biolabs, Beverly, MA, USA) and separated on 3% agarose gel (Figs 1A and 2A). The 829C allele had one Bgl*I* restriction site that resulted in two bands (112 bp and 25 bp) and the 20541C allele had one Ase*I* restriction site that resulted in two bands of 82 bp and 21 bp. Genotyping was carried out without knowledge of the case-control status of the subjects. The *CASP3* 829 A>C and 20541 C>T genotypes revealed by PCR-RFLP analysis were further confirmed by direct DNA sequencing (Figs 1B and 2B). Ten percent of the samples were randomly selected for repeated assays, and the results were 100% consistent.

### Construction of promoter-reporter plasmids

To verify whether the 829 A>C SNP influences the transcriptional activity of CASP3, The primers used for amplifying CASP3 promoter were 5’-ata cGCTAGCTACCCAGT GACCAG CAAGTG-3’and 5’-gataAAGCTTGGTGG CAAAACAAACACTCC-3’, which contain Nhe *I* and Hind *III* (NEB, MA, USA) cloning sites (underlined sequences), respectively [13]. The resulting PCR product were subsequently digested with Nhe *I* and Hind *III* and cloned into the pGL3-basic vector (Promega, Madison, USA) containing the firefly luciferase gene as a reporter. To produce the luciferase construct containing the 829A allele, a pair of primers 5’-GGT TTAAAC TCCAATTCATTT TCGGCC C-3’ and 5’-GAA TTG GAGTTTAAACCC TGCAACTATCTC-3’ was used to make the single site mutagenesis (Invitrogen, Carlsbad, CA, USA). The constructs were all confirmed by DNA sequencing.

### Cell culture, transfection and luciferase assay

Human lung cancer cells (A549, NCI-H1975) used for the luciferase reporter analysis were provided from Cobioer Biosciences (Cobioer, Nanjing, China). A549 and NCI-H1975 lung cells were grown in RPMI 1640 medium supplemented with 10% fetal bovine serum (FBS) (GIBCO, NY, USA) and 1% of penicillin and streptomycin in a humidified environment at 37°C with 5% CO_2_. Cells (2×10^5^) were plated in a 24-well plate and grown to 80–90% confluence. Cells were co-transfected with 1ug firefly luciferase reporter plasmid and 1ng pRL-SV40 (Promega, Madison, USA) using Lipofectamine 2000 reagent (Invitrogen, CA, USA). Luciferase activity was determined according the manufacturer’s protocol using a dual-luciferase reporter assay system (Promega, Madison, USA). For each plasmid construct, three independent transfection experiments were carried out, and each luciferase assay was performed in triplicate. The empty pGL3 Basic vector cotransfected with pRL-SV40 plasmid served as a control. Fold increase was calculated by defining the activity of empty pGL3 Basic vector as 1. Differences were determined by *t* test, and *P* .01 was considered significant.

### Statistical analysis

Student’s t-test was used to compare the means of age andχ^2^-test was used to compare the frequency distributions among cases and controls. Odds ratios (OR) and 95% confidence intervals (CI) were computed to evaluate the susceptibility of lung cancer using multivariate logistic regression analysis adjusted by age, sex, and smoking. All statistical tests were two-sided, and a *P* -value of <0.05 was considered significant using Statistical Analysis System software (Version 16.0; IBM, New York, USA).

## Results

### Subject characteristics

The characteristics of the two study groups were summarized in Table 1. No statistically significant differences in terms of age and gender distributions were found between the cases and controls. However, more smokers were present among lung-cancer patients than that among cancer-free controls, and the ratios respectively are 61.4% and 48.4% (*P* < 0.001). Moreover, there were 67.6% smokers over 28 pack-years in the case group and just 41.1% over 28 pack-years smokers in control group, showing that smoking causes the majority of lung cancers in the participants. Based on the pathological types of cases, 43.0% belonged to squamous-cell carcinoma, 50.4% belonged to adenocarcinoma, and 6.6% belonged to other types, including undifferentiated cancer, bronchi alveolar carcinoma, and mixed-cell carcinoma.

**Table 1:** Frequency distribution of select characteristics by case-control status.

Variable	Cases		Controls		*P* value*
	N = 1000		N = 1000		
	No.	%	No.	%	
Sex					1.000
Male	712	71.2	712	71.2	
Female	288	28.8	288	28.8	
Age					1.000
≤ 50	213	21.3	213	21.3	
51–60	363	36.3	363	36.3	
> 60	424	42.4	424	42.4	
Smoking status					< 0.001
Non-smoker	386	38.6	516	51.6	
Smoker	614	61.4	484	48.4	
Pack-years					0.001
<16	98	16	148	30.6	
16–28	101	16.4	137	28.3	
> 28	415	67.6	199	41.1	
Histological type^#^					
SC	430	43.0			
AC	504	50.4			
Others^#^	66	6.6			

^*^ two-sided χ^2^ test

^#^ SC: Squamous cell carcinoma; AC: adenocarcinoma; others: adenosquamous carcinoma (n = 7), undifferentiated cancer (n = 56), and large cell carcinoma (n = 3).

### Association of CASP3 genotypes with lung cancer risk

Table 2 displayed that the genotype distributions of *CASP3* 829 A>C and 20541 C>T in the cases and controls respectively. All observed genotype frequencies of 829 A>C and 20541 C>T in the controls conform to Hardy Weinberg equilibrium (*P* = 0.915 and *P* = 0.078, respectively). Compared with the individuals with 829AA genotype, the individuals with at least 829 C allele had remarkably increased risk of lung cancer (OR (95% CI) = 1.33 (1.09–1.63) vs. 1.55 (1.19–2.01), respectively). No significant changed risk of lung cancer was found to relate to the 20541 C>T genotype. The OR (95% CI) was 1.18 (0.97–1.44) and 1.16 (0.86–1.57) for the carriers with 20541 CT and 20541 TT genotypes, respectively.

**Table 2:** Genotype distribution of *CASP3* in cases and controls and their associations with the risk of lung cancer.

Genotype variants	Controls	Cases	OR (95%CI)^*^	*P* value
	(N = 1000)	(N = 1000)		
	No. (%)	No. (%)		
829 A/C				
AA	355(35.5)	290(29.0)	1.00 (ref.)	
AC	483(48.3)	508(50.8)	1.33(1.09–1.63)	0.006
CC	162(16.2)	202(20.2)	1.55(1.19–2.01)	0.001
20541 C/T				
CC	596(59.6)	574(57.4)	1.00 (ref.)	
CT	294(29.4)	321(32.1)	1.18(0.97–1.44)	0.107
TT	110(11.0)	105(10.5)	1.16(0.86–1.57)	0.340

^*^Data were calculated by unconditional logistic regression and adjusted for sex, age and smoking status

### Interaction of CASP3 Genotypes and gender, age and classification of lung cancer

The factors including age, gender and smoking status were selected to identify whether *CASP3* 829A>C and 20541 C>T polymorphisms had some relations with lung cancer. We operated a multivariate regression model to obtain the association between *CASP3* 829A>C and 20541 C>T genotypes and risk of lung cancer with adjustment for age, gender and smoking status. The results were shown in Table 3. In the subgroups of male group, *CASP3* 829 AC or CC genotype evidently increased the riskof lung caner, with OR (95% CI) of 1.42 (1.11–1.81) and 1.51 (1.11–2.05), respectively, and also in younger group, with OR (95% CI) of 2.73 (1.71–4.34) and 4.02 (2.20–7.32), respectively, compared with the common homozygous 829 AA genotype. In the mean time, we compared the risk of lung cancer associated with the *CASP3* 829A>C polymorphisms and different histological types of lung cancer, including SCC, adenocarcinoma, and other histological types. We found that 829AC or 829CC genotype increased adenocarcinoma risk compared with the AA genotype with OR (95%CI) of 1.33 (1.04–1.70) and 1.51(1.09–2.07). We haven’t obvienced any evidence for conjectural interaction between the 20541 C>T variant and these selected variables (Table 4).

**Table 3:** CASP3 829 A>C genotype frequencies in cases of lung cancer and controls, stratified by age, sex and classifications of lung cancer.

Variables	Genotypes	Cases/Controls	OR (95%CI)	*P* value
Sex				
Male	AA	205/255	1.00 (ref.)	
	AC	356/333	1.42 (1.11–1.81)	0.005
	CC	151/124	1.51 (1.11–2.05)	0.010
Female	AA	85/100	1.00 (ref.)	
	AC	152/150	1.18 (0.82–1.71)	0.377
	CC	5138	1.48 (0.88–2.48)	0.136
Age				
≤50	AA	38/84	1.00 (ref.)	
	AC	124/101	2.73 (1.71–4.34)	< 0.001
	CC	51/28	4.02 (2.20–7.32)	< 0.001
51–60	AA	102/127	1.00 (ref.)	
	AC	190/179	1.33 (0.96–1.86)	0.089
	CC	71/57	1.61(1.04–2.50)	0.033
>60	AA	150/144	1.00 (ref.)	
	AC	194/203	0.96 (0.70–1.33)	0.803
	CC	80/77	0.96 (0.63–1.44)	0.832
Histological type^*^				
SC	AA	129/355	1.00 (ref.)	
	AC	207/483	1.29 (0.98–1.70)	0.073
	CC	94/162	1.55 (1.10–2.20)	0.013
AC	AA	144/355	1.00 (ref.)	
	AC	263/483	1.33 (1.04–1.70)	0.024
	CC	97/162	1.51 (1.09–2.07)	0.012
Other	AA	17/355	1.00 (ref.)	
	AC	38/483	1.64 (0.91–2.95)	0.102
	CC	11/162	1.48 (0.67–3.24)	0.329

^*^SC: Squamous cell carcinoma; AC: Adenocarcinoma; others: adenosquamous carcinoma (n = 7), undifferentiated cancer (n = 56), and large cell carcinoma (n = 3)

**Table 4:** Risk of lung cancer association with *CASP3* 829 A>C genotypes by smoking status.

Smoking status	CASP3 829 A>C genotype								
	AA^*^	OR (95%CI)^#^	*P* value	AC^*^	OR (95%CI)^#^	*P* value	CC^*^	OR (95%CI)^#^	*P* value
Non-smoker	102/181	1.00 (ref.)		124/101	1.48(1.08–2.02)	0.014	51/28	1.64(1.08–2.20)	0.018
Smoker	188/174	2.60(1.78–3.78)	< 0.001	296/227	2.68(1.89–3.81)	< 0.001	130/83	3.23(2.12–4.92)	< 0.001
< 16 pack-years	32/58	1.30(0.77–2.21)	0.333	46/62	1.82(1.11–2.99)	0.018	20/28	2.01(1.02–3.97)	0.044
16–28 pack-years	33/54	1.57(0.91–2.71)	0.104	48/60	2.57(1.50–4.42)	0.001	20/23	2.83(1.38–5.80)	0.004
> 28 pack-years	123/62	4.64(2.96–7.19)	< 0.001	202/105	4.95(3.22–7.61)	< 0.001	90/32	7.29(4.23–12.59)	< 0.001

^*^ Number of cases/number of controls

^#^Adjusted for age and sex

### Interaction of *CASP3* Genotypes and smoking

Because Tobacco smoking is an accepted aetiological predisposition for lung cancer, we investigated the interaction between the CAPS3 polymorphisms and smoking, which was shown in Tables 5 and 6. We achieved that the *CASP3* 829AC or CC genotype compared with the AA genotype was associated with increased risk of lung cancer in non-smoker group (OR = 1.48, 95%CI = 1.08–2.02; OR = 1.64, 95%CI = 1.09–2.48). Moreover, these variant genotypes were significantly associated with a two- or three-fold increased risk of lung cancer in smokers (OR = 2.68, 95%CI = 1.89–3.81; OR = 3.23, 95%CI = 2.21–4.92). Among carriers with 20541CT genotype, the ORs (95%CI) of risk with lung cancer for smoking <16, 16–28, or > 28 pack-years were 1.16(0.65–2.07), 1.66(0.98–2.82) and 5.01(3.31–7.58) compared with the 20541CC carriers. And among carriers with 20541CT genotype, the ORs (95%CI) were 0.86(0.33–2.20), 2.12(0.83–5.41) and 5.71(2.68–12.16).

**Table 5:** CASP3 20541 C>T genotype frequencies in cases of lung cancer and controls, stratified by age, sex and classifications of lung cancer.

Variables	Genotypes	Cases/Controls	OR (95%CI)	*P* value
Sex				
Male	AA	413/438	1.00 (ref.)	
	AC	232/208	1.19 (0.94–1.51)	0.151
	CC	67/66	1.29 (0.88–1.89)	0.196
Female	AA	161/158	1.00 (ref.)	
	AC	89/86	0.97 (0.67–1.43)	0.889
	CC	38/44	0.85 (0.50–1.43)	0.530
Age				
≤50	AA	100/105	1.00 (ref.)	
	AC	75/60	1.27 (0.82–1.98)	0.289
	CC	38/48	0.75 (0.44–1.28)	0.291
51–60	AA	208/211	1.00 (ref.)	
	AC	118/116	1.04 (0.76–1.44)	0.810
	CC	37/36	1.06 (0.64–1.76)	0.805
>60	AA	266/280	1.00 (ref.)	
	AC	128/118	1.18 (0.76–1.44)	0.317
	CC	37/36	1.20 (0.67–2.15)	0.545
Histological type^*^				
SC	AA	266/596	1.00 (ref.)	
	AC	122/294	0.99 (0.75–1.30)	0.950
	CC	42/110	1.47 (0.95–2.28)	0.082
AC	AA	284/596	1.00 (ref.)	
	AC	173/294	1.24 (0.97–1.57)	0.082
	CC	47/110	0.85 (0.58–1.24)	0.391
Other	AA	24/596	1.00 (ref.)	
	AC	26/294	2.24 (1.26–3.99)	0.006
	CC	16/110	0.61 (1.91–7.77)	0.001

^*^SC: Squamous cell carcinoma; AC: Adenocarcinoma; others: adenosquamous carcinoma (n = 7), undifferentiated cancer (n = 56), and large cell carcinoma (n = 3)

**Table 6:** Risk of lung cancer association with *CASP3* 20541 C>T genotypes by smoking status.

Smoking status	CASP3 20541 C>T genotype								
	CC*	OR (95%CI)^#^	*P* value	CT*	OR (95%CI)^#^	*P* value	TT*	OR (95%CI)^#^	*P* value
Non-smoker	201/289	1.00(ref.)		127/153	1.10(0.81–1.50)	0.532	58/74	0.92(0.60–1.40)	0.688
Smoker	373/307	2.06(1.58–2.68)	< 0.001	194/141	2.68(1.91–3.76)	< 0.001	47/36	2.48(1.49–4.14)	< 0.001
< 16 pack-years	65/95	1.17(0.80–1.70)	0.428	26/37	1.16(0.65–2.07)	0.625	7/16	0.86(0.33–2.20)	0.745
16–28 pack-years	54/85	1.12(0.75–1.69)	0.584	36/43	1.66(0.98–2.82)	0.060	11/9	2.12(0.83–5.41)	0.116
> 28 pack-years	254/127	3.32(2.45–4.51)	< 0.001	132/61	5.01(3.31–7.58)	< 0.001	29/11	5.71(2.68–12.16)	< 0.001

^*^ Number of cases/number of controls

^#^Adjusted for age and sex

### Effects of CASP3 829 A>C SNP on the transcriptional activity

**Fig 3:**
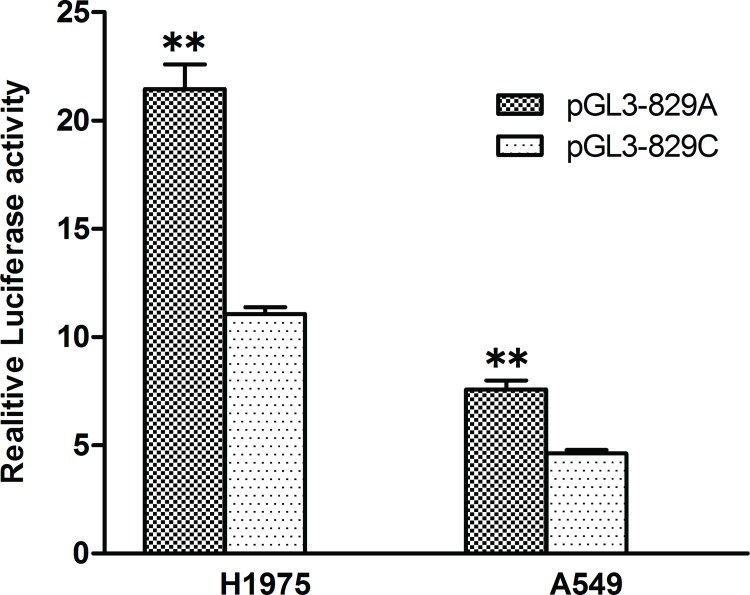
Transcription activity analysis of the *CASP3* 829 A>C variant in A549 and NCI-H1975 cells. Luciferase activity profiles were assayed following transfection of the constructs into A549 and NCI-H1975. pGL3-829A and pGL3-829C denote caspase-3 promoter constructs containing the 829A or 829C allele, respectively. All of the constructs were cotransfected with pRL-SV40 to standardize the transfection efficiency. Values were means±SD from more than 3 separate experiments that were each performed in triplicate. **P < .001 compared with each of the construct counterparts.

To explored the possible impact of 829 A>C SNP on *CASP3* transcriptional activity, we constructed promoter vectors containing 829A or 829C alleles and dual luciferase assay was carried out in lung cancer cell lines, A549 and NCI-H1975. Report gene expressions driven by 829A containing CASP3 promoter were 1.64-fold, 1.94-fold greater than those driven by CASP 829C containing counterparts in A549 and NCI-H1975 cells (P<0.001) (Fig 3). These results suggest that the 829 A>C polymorphism influences CASP3 promoter activity.

## Discussion

In the previous study, we reported that FAS/FASL, as another apoptosis associated protein, was associated with the risk of esophageal cancer[14]. In the current study, we investigated the potential association of *CASP3* polymorphisms (829 A>C and 20541 C>T) and with the risk of lung cancer in Chinese population. We observed that individuals who carried *CASP3* 829C allele were at significantly increased risk for lung cancer. Moreover, the risk was more evident in the subgroups of male subjects, younger subjects and smokers. These results are consistent with our previous study for esophageal squamous cell carcinoma [14]. This finding further provided evidences that the CASP3 played a significant role in human carcinogenesis.

By means of eliminating DNA-damaged cells, apoptosis can keep hosts cells away from cancer development [15]. Caspases are important mediators of apoptosis. CASP3, as an execution-phase caspase, is known to play a crucial role during apoptosis. Some research demonstrated the mutation of *CASP3* existed in human tumor tissues and cell lines as expected [9, 10, 16]. Other studies demonstrated that CASP3 was essential to the regulation of B-cell homeostasis by the way of DNA fragmentation, chromatin margination, nuclear collapse and cleavage of many key participators involved in apoptosis [17–21]. Importantly, one of the essential features of tumor cells is the ability of cells to avoid apoptosis, and this capability can help them destroy the anticancer defense mechanisms[22]. Mandruzzato and Wang have reported that a large number of caspases mutations in human tumor cells, which caused reduced apoptotic activities [23, 24]. It suggested that the mutation of *CASP3* was particularly prone to occur in human cancer tissues, or *CASP3* mutation genotype resulted in carcinogenesis. Moreover, in many tumors, it was probably that an analogous loss or down regulation of CASP3 expression may happen [8, 25, 26]. It is possible that this *CASP3* mutation may have resulted that the target tissue operated the apoptosis disadvantageously and thus raised the potential risk of carcinogenesis. This presence indicates that the *CASP3* gene mutates in human cancer on occasion.

One study determined nine potentially functional polymorphisms in the *Caspase* on survival of early-stage NSCLC patients. Their conclusion was that the *CASP7* rs2227310 and *CASP9* rs4645981 polymorphisms may affect survival in early-stage NSCLC [27]. Some association studies have suggested possible links between *CASP3* polymorphism and the susceptibility to several of cancers, including endometrial cancer, prostrate cancer and head and neck cancer [28–30]. One case-control study was demonstrated that *CASP3* rs4647601 TT genotype was related with an increased dangerous impact of squamous cell carcinoma of the Head and Neck [29]. A meta-analysis showed that the homozygote (CC) of rs2705897 (A/C) in the *CASP3* gene had a positive association with cancer susceptibility [31]. In addition, Jang et al. demonstrated that individuals carried at least one variant allele of the *CASP3* -928A>G, 77G>A, and 17532A>C polymorphisms contributed to the genetic susceptibility to lung cancer [32]. Similarity, our present study showed that functional *CASP3* 829 A>C polymorphism, another significant SNP of *CASP3*, increased the susceptibility of lung cancer. Our previous real-time PCR analyses indicated that individuals carried *CASP3* 829AA genotype had obviously higher RNA levels than that carried 829 AC and 829 CC genotypes [12]. This is consistent with our reporter gene results in lung cancer cells. We found that *CASP3* 829A allele containing promoter had higher reporter gene transcriptional activity than 829C allele containing *CASP3* promoter. Evasion of apoptosis is a common feature of malignancy. The acquired ability to resist apoptotic stimuli is one of the primary characteristics of a malignant cell. This result implied that the 829 A>C polymorphism caused the decline of *CASP3* transcriptional activity and further contribute to the increased risk of developing lung cancer.

Our findings of a significantly elevated risk, most evident in male and younger subjects with a tendency of increased risk with more variant alleles, suggested that for genetic susceptibility the *CASP3* SNPs might be typical markers for lung cancer, because characteristics of genetic susceptibility include an early age of lung cancer onset. It is also possible that gender was important for lung cancer susceptibility. Quite a lot studies showed that gender is an independent or interactional factor for lung cancer susceptibility [33]. Interestingly, another finding of our study *CASP3* 829C allele was associated with higher risk of lung cancers, suggesting that these polymorphisms might be ecumenical risk causation for most of frequent tumor. These results are accordance with our previous conclusion of esophageal squamous cell carcinoma. All these findings indicated that apoptosis- interrelated *CASP3* is a cancer susceptibility gene and plays an important role in human carcinogenesis.

Because tobacco smoking is a proverbial etiological factor for many kinds of cancer including lung cancer, it has been shown to mediate an alteration of the intracellular balance between pro- and anti-apoptogenic factors [34]. An analysis of *CASP9* promoter polymorphisms contributing to genetic susceptibility to lung cancer suggested that *CASP9* polymorphisms and their haplotypes interacted with tobacco smoking[35]. One of our published study observed that tobacco smoking worsened the trend of the susceptibility of lung cancer by genetic variant[36]. Therefore we investigated gene-environment interaction between the *CASP3* polymorphisms and smoking. An important point of our study was *CASP3* polymorphisms had an interaction with cigarette. The *CASP3* 829CC genotype modified the susceptibility of lung cancer in habitual smokers but outside non-smokers, especially in heavy smokers with OR (95%CI) of 7.29 (4.23–12.59), suggesting a gene-environment interaction. We also found the increased risk of lung cancer among both all the smoker group and heavy smoker subgroup, who took with the *CASP3* 20541 CT and TT genotype, however the similar significant association with incremental risk of lung cancer was not proved in nonsmoker, different gender and age group.

In summary, we identified that two SNPs of *CASP3* gene increased the susceptibility of lung cancer with a large sample size in this case-control study. Tabaco smoking modulated and was interacted the association with *CASP3* polymorphisms to lung cancer. The present results are in line with our prior conclusions in the esophageal squamous cell carcinoma research; further indicating that apoptosis-interrelated *CASP3* is a cancer susceptibility gene and plays a significant role in human carcinogenesis.
